# Routine Serial Follow-up Blood Cultures After a Negative Result Within 48 Hours of Methicillin-susceptible *Staphylococcus aureus* Bacteremia: Low Yield, High Cost

**DOI:** 10.1093/cid/ciag418

**Published:** 2026-07-15

**Authors:** Marianne M C Hendriks, Eline H H M Kamps, Cairo Norberhuis, Mihai G Netea, Matthijs S Berends, Ianthe Maat, Sander van Assen, Jaap ten Oever, Ilse J E Kouijzer

**Affiliations:** Department of Internal Medicine and Radboud Community for Infectious Diseases, Radboud University Medical Center, Nijmegen, The Netherlands; Department of Internal Medicine and Radboud Community for Infectious Diseases, Radboud University Medical Center, Nijmegen, The Netherlands; Department of Internal Medicine, Treant, Emmen, The Netherlands; Department of Internal Medicine and Radboud Community for Infectious Diseases, Radboud University Medical Center, Nijmegen, The Netherlands; Department of Immunology and Metabolism, Life and Medical Sciences Institute, University of Bonn, Bonn, Germany; Department of Medical Epidemiology, Certe Foundation, Groningen, The Netherlands; Department of Medical Microbiology and Infection Prevention, University Medical Center Groningen, Groningen, The Netherlands; Department of Medical Microbiology and Radboud Community for Infectious Diseases, Radboud University Medical Center, Nijmegen, The Netherlands; Department of Internal Medicine, Treant, Emmen, The Netherlands; Department of Internal Medicine and Radboud Community for Infectious Diseases, Radboud University Medical Center, Nijmegen, The Netherlands; Department of Internal Medicine and Radboud Community for Infectious Diseases, Radboud University Medical Center, Nijmegen, The Netherlands

**Keywords:** *Staphylococcus aureus* bacteremia, skip phenomenon, early skip phenomenon, follow-up blood cultures

## Abstract

**Background:**

Follow-up blood cultures (FUBCs) are essential in managing *Staphylococcus aureus* bacteremia (SAB). Skip phenomenon (SP), intermittent negative cultures despite ongoing bacteremia, occurs in 4%–13% of SAB patients and may lead to premature cessation of diagnostics and inappropriate treatment. Early SP, defined as negative FUBCs within 48 hours after index culture followed by subsequent positivity, is poorly characterized but concerning, as it can mask persistent bacteremia (PB) linked to worse outcomes.

**Methods:**

We conducted a cohort study at 2 Dutch hospitals, including adult SAB patients. Patients were categorized as having early clearance (EC), PB, or SP. Primary outcome was early SP prevalence, secondary outcomes included the proportion of potentially missed cases of PB when relying on 1 negative FUBC within 48 hours, reasons for obtaining FUBCs in early SP patients, and estimated costs of unnecessary FUBCs.

**Results:**

Among 1005 included SAB patients, 31 patients had early SP (3.1%). In 27 of these (87%), clinical condition prompted repeat FUBCs. If 1 negative FUBC within 48 hours was considered sufficient, 4 patients (1%) without clinical triggers would have been misclassified as EC, potentially missing PB. Potential cost savings from discontinuing FUBCs after 1 negative result within 48 hours were 13 513 USD per 100 patients.

**Conclusions:**

Early SP in SAB was uncommon. Clinically stable, afebrile patients without signs of metastatic infection and 1 negative FUBC within 48 hours have a very low risk of missed PB. A day-2 focused, clinically guided approach may reduce low-yield testing and costs, while maintaining patient safety.

## Teaser

Early skip phenomenon in *Staphylococcus aureus* bacteremia is rare (3.1%) with a very low risk of missed persistent bacteremia (1%). A practical day-2 focused, clinically guided strategy for follow-up blood cultures reduces low-yield testing and costs, while maintaining patient safety.

**(See the Editorial Commentary by Martin and Tong on pages e754–6.)**

Bacteremia duration is a key prognostic factor in *Staphylococcus aureus* bacteremia (SAB): persistent bacteremia (PB) often reflects persistent infectious foci, such as undrained abscesses or intravascular infections [1–3]. PB is associated with higher infection-related mortality [4], requiring more extensive clinical, microbiological and diagnostic evaluation [5]. Therefore, follow-up blood cultures (FUBCs) in patients with SAB are an important component of patient management. Guidelines recommend obtaining FUBCs every 2–4 days until bacteremia clearance [3, 6]. However, debate continues whether a single negative FUBC reliably indicates bloodstream clearance due to the “skip phenomenon (SP),” where intermittent negative blood cultures occur despite PB [7–12]. Recent studies therefore recommend at least 2 consecutive days of negative FUBC sets to confirm bacterial clearance and accurately determine bacteremia duration [7–12].

The SP occurs in 4%–13% of SAB patients [7–12]. Proposed mechanisms include low or fluctuating bacterial load, timing of sampling relative to intermittent bacterial release, blood collection volume, and partial or complete suppression by antibiotic therapy. These can lead to false-negative cultures and erroneously presumed bacteremia clearance, potentially leading to premature cessation of FUBC collection, incomplete source evaluation, or inappropriate de-escalation or shortening of therapy. Although SP appears to occur in patients with risk profiles similar to those with PB and may carry comparable adverse outcomes [1, 7, 9], it remains insufficiently characterized regarding its occurrence early in the course of infections. Early SP, defined as an intermittent negative blood culture within 48 hours of the index culture, is particularly concerning. In contrast to intermittent negative FUBCs later in the disease course, when PB criteria is already recognized and prompts further diagnostic evaluation [5], early negative FUBCs may lead clinicians to mistakenly assume bacteremia clearance, potentially resulting in insufficient diagnostics and interventions as well as premature de-escalation of therapy [5, 7, 13, 14].

Therefore, we examined the prevalence of early SP in SAB, the clinical significance of early SP in SAB, whether a single negative FUBC early during SAB is safe to presume clearance, and cost savings associated with this approach.

## METHODS

### Study Design

We performed a retrospective multicenter cohort study at 2 large hospitals in the Netherlands, 1 university hospital and 1 nonuniversity teaching hospital. We obtained approval from the research ethics boards of both hospital sites (2025–18003). All adults with SAB between January 2013 and May 2025 were eligible for inclusion. Supplementary Table 1 provides the exact timeframes per hospital. SAB was defined as 1 or more blood culture bottles positive for *S. aureus*. Exclusion criteria were: polymicrobial bacteremia (except when coisolated microorganisms were common skin contaminants) or contaminated blood cultures (as judged by an infectious diseases specialist or clinical microbiologist, with no antibiotics initiated, and no recurring SAB episode), immediate transfer to another hospital, and death or transition to palliative care ≤72 hours after the index blood culture to minimize the potential impact of early mortality on classification of early SP or EC. Because early SP and EC occur within the first 2 days after the index blood culture, patients who died before this time window would not have had the opportunity for adequate assessment of these outcomes. In addition, patients with fewer than 2 days of FUBCs in the first 14 days following SAB were excluded, as ≥2 days of FUBCs were required to reliably determine the presence or absence of SP. SAB caused by methicillin-resistant *S. aureus* (MRSA) was also excluded, since the prevalence of MRSA in our country is extremely low [15], and therefore too small to allow meaningful or reliable analyses of SP occurrence within this subgroup. In patients with multiple episodes of SAB, only the first episode was included for analysis. Follow-up was 90 days after the first day of SAB to assess all-cause mortality and relapse/reinfection. Thirty-day mortality was used as a proxy for infection-related mortality [4].

### Data Collection and Definitions

Clinical data were manually retrieved from electronic medical records and included demographics, comorbidities [16], laboratory results, microbiological data, imaging, and antimicrobial treatment. A FUBC was defined as 2 blood culture bottles collected during a single peripheral venipuncture or central venous catheter aspiration. Blood cultures were performed using the BacT/ALERT system (bioMérieux, France) or BD BACTEC™ FX system (using BD BACTEC™ Plus Aerobic Medium and BD BACTEC™ Plus Anaerobic medium bottles), depending on hospital site (Supplementary Table 1). Species identification was performed using MALDI-TOF. The collected blood cultures were promptly transported to the microbiology laboratory, and immediately set up in the BacT/ALERT of BD BACTEC™ system, respectively. When blood cultures were positive, the time to positivity (TTP) was recorded. TTP was defined as the time in hours from start of incubation of the blood culture until the first blood culture bottle became positive. TTP data became available only in the later years of the study, time frames for each hospital site are provided in Supplementary Table 1.

Bacteremia duration was calculated from the index blood culture to the date of the final positive blood culture in days [17]. A bacteremia duration of less than 48 hours after the initial positive blood culture was considered early clearance (EC). EC was defined as a day of negative FUBCs obtained within 48 hours of the index blood culture or, if unavailable within this window, negative results on the subsequent FUBCs. Because bacteremia duration was determined by the timing of the last positive blood culture, a first negative FUBC obtained after 2 days was still considered compatible with early clearance. PB was defined as the presence of positive FUBCs at least 48 hours after the index blood culture [2, 18]. SP was defined as at least 1 calendar day with negative FUBCs after the index culture, followed by at least 1 new positive blood culture for *S. aureus.* Patients were classified as early SP if this phenomenon occurred within 48 hours of the initial positive culture (Figure 1). All abbreviations are listed in Table 1.

**Figure 1.**
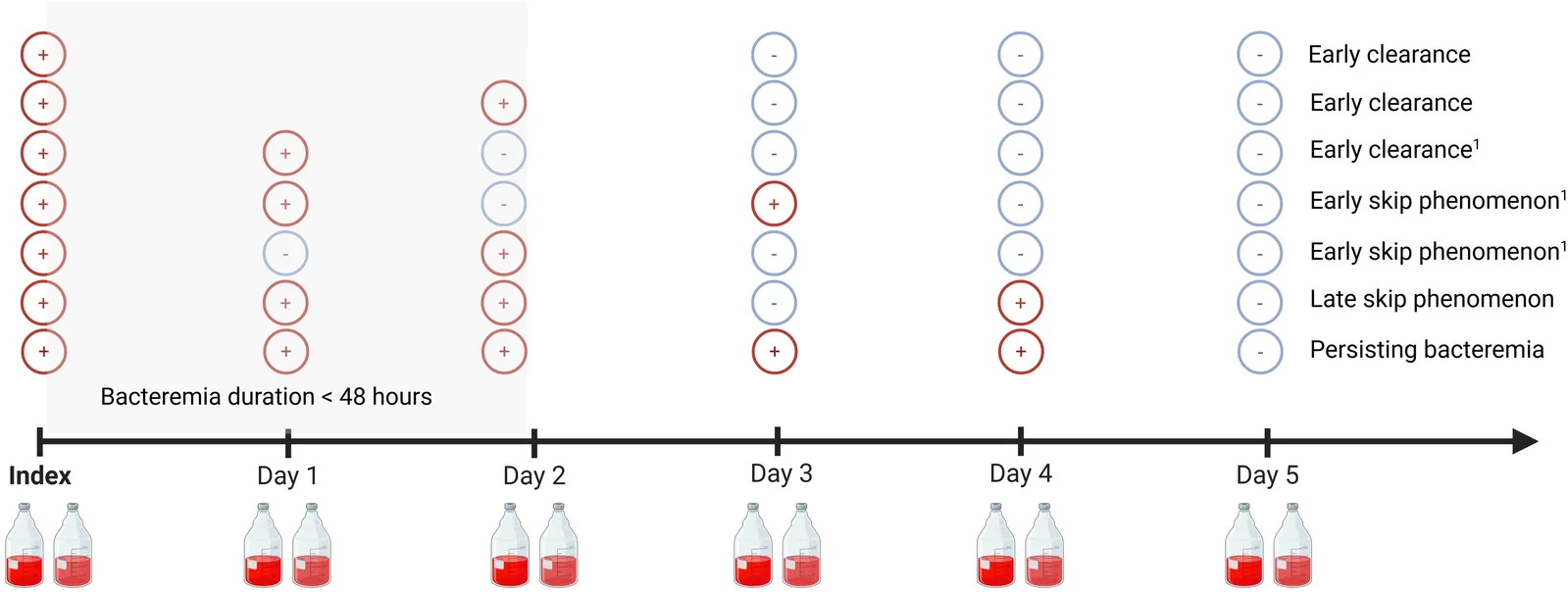
Schematic representation of early clearance, early skip phenomenon, late skip phenomenon, and persistent bacteremia. Plus sign indicates a blood culture positive for *S. aureus*; negative sign indicates a blood culture negative for *S. aureus*. ^1^Included in FUBC cost analysis. Abbreviation: S, *Staphylococcus*. Figure created in BioRender. Hendriks, M. (https://BioRender.com/iubhkb1) is licensed under CC BY 4.0.

**Table 1.** List of Abbreviations and Definitions

Abbreviation	Definition
Early SP	Early skip phenomenon: patients with skip phenomenon within 48 h of the initial positive culture
EC	Early clearance: bacteremia duration of less than 48 h after the initial positive blood culture
FUBC	Follow-up blood cultures: either 1 or 2 blood culture bottles collected during a single peripheral venipuncture or vascular access aspiration
PB	Persistent bacteremia: presence of positive follow-up blood cultures at least 48 h after the index blood culture
SAB	*Staphylocccus aureus* bacteremia: one or more blood culture bottles positive for *S. aureus*
SP	Skip phenomenon: at least 1 calendar day with negative follow-up blood cultures after the index culture, followed by at least 1 new positive blood culture for *S. aureus*
TTP	Time-to-positivity: the time in hours from start of incubation of the blood culture until the first blood culture bottle became positive
Unnecessary FUBCs	Unnecessary follow-up blood cultures: FUBCs obtained after the first documented negative FUBC within the initial 48 h, in the absence of clinical indication suggestive of persistent infection

The index blood culture date was considered as day 0. FUBCs were considered clinically indicated in case of persistent or recurrent fever, hemodynamic instability and/or ICU admission, presence of intravascular- and intracardiac-prosthetic material, unresolved focus of infection, and confirmed (intravascular) metastatic infection [19]. Unnecessary FUBCs were defined as FUBCs obtained after the first documented day of negative FUBC within 48 hours after SAB onset, in the absence of a clinical indication suggestive of persistent infection. The onset of SAB was categorized as community-acquired, healthcare-associated, or hospital-acquired [1]. Metastatic SAB was defined as bacteremia with a site of infection remote from the primary focus caused by hematogenous seeding or extension of infection beyond the primary focus [1].

### Outcome Measures

The primary outcome was the prevalence of early SP in SAB. Secondary outcomes included the proportion of potentially missed PB cases due to early SP, reasons for FUBCs in early SP patients, the diagnostic performance (accuracy, sensitivity, and negative predictive value [NPV]) of FUBCs over time and costs associated with unnecessary FUBCs.

### FUBCs-analysis

We identified the reasons for performing additional FUBCs after an initial negative result in patients with EC and early SP. To estimate the proportion of potentially missed cases of PB due to early SP, we calculated the percentage of SP patients with negative FUBCs within 48 hours who did not meet the clinical criteria for FUBCs.

To evaluate the reliability of FUBCs over time, we analyzed trends in diagnostic accuracy during the first 14 days after SAB diagnosis using the approach described by Stewart et al, incorporating all FUBC results obtained within this 14-day time period [10]. Individual FUBC results were classified using standard definitions: a positive FUBC indicated ongoing bacteremia confirmed by a positive culture; a false negative was defined as a negative FUBC both preceded and followed by a positive culture for *S. aureus* within the first 14 days of SAB, indicating SP. FUBCs were considered false negative only when all sets collected within a calendar day were negative. A true negative FUBC denoted sustained clearance with no subsequent positive cultures; if no subsequent FUBCs were obtained after a single negative FUBC, the negative FUBC was considered true negative. Accuracy was calculated as the ratio of positive and true negative FUBC to the total number of FUBC. Sensitivity was calculated as the proportion of positives out of the total of positive and false negative FUBCs. The NPV was calculated as the ratio of true negatives to all negative results to assess the reliability of culture results over time.

### Costs

Microbiological testing costs were estimated based on the specific brand and model of culture plates utilized during the study period, using price data provided by the microbiology laboratories. All costs were converted to U.S. dollars (USD) according to the average exchange rate for the corresponding timeframe. First, we calculated the costs as they were actually incurred in the clinical care of this cohort. We then determined the incremental costs attributable to each unnecessary negative FUBC obtained during the study period, in order to quantify the economic impact of unnecessary FUBC in routine care. Potential savings were expressed as USD per 100 patients.

### Statistical Analysis

Descriptive statistics were performed to summarize baseline characteristics. Continuous variables were presented as medians with interquartile ranges (IQRs). Categorical variables were presented as frequencies and percentages.

Group comparisons used the Kruskal–Wallis or Mann–Whitney *U* test for continuous variables and the *χ*^2^ or Fisher's exact test for categorical variables. In cases with <5 observations, the Monte Carlo simulation was applied. Missing data were not imputed. A formal competing-risk analysis for death was not applied, since all included patients survived long enough and had sufficient microbiological follow-up to allow accurate evaluation of early SP and EC. A sensitivity analysis was performed excluding patients with a single day of negative FUBC and no subsequent FUBCs, in order to assess the diagnostic accuracy of FUBCs and calculate the proportion of missed PB in patients with a negative FUBC within 48 hours, whose FUBCs remained negative thereafter. A subgroup analysis was performed to assess the prevalence of EC, SP, and PB in patients with and without relevant prosthetic material in situ. Statistical significance was tested at a 2-sided *P* value of .05, and 95% confidence intervals (CIs) were reported for all inferential statistics. All analyses were conducted using IBM SPSS Statistics Software for Windows, Version 29.0.2.0 (IBM Corp., Armonk, NY, USA).

## RESULTS

### Patient and FUBC Characteristics

Between January 2013 and March 2025, 1386 adult patients were diagnosed with SAB, of whom 1005 met the inclusion criteria (Figure 2). A total of 10 510 blood cultures were obtained from 1005 patients within the first 14 days after SAB onset (Table 2). SP occurred in 69 patients (6.9%), 709 patients were classified as having EC (70.5%) and 227 as having PB (22.6%). Patients with intravascular or intracardiac-prosthetic material in situ did not have higher prevalences of PB or SP (Supplementary Table 2). Among the 751 patients who underwent FUBCs within 2 days, 391 (52.1%) had negative FUBCs during this time period; 166 of these patients did not have clinical indications for FUBCs after the negative results within 48 hours. The proportion of positive cultures declined steadily over time, indicating effective clearance during therapy: 67.8% on day 1 versus 2.0% on day 14 (Figure 3). False-negative results occurred in 89 cultures among 69 patients, peaking between days 2 and 4. The NPV of FUBCs increased over time: 0.92 to 0.96 between days 1 and 4, respectively, and consistently exceeding 0.98 from day 5 onward, reflecting greater diagnostic reliability at later stages. A sensitivity analysis by excluding 69 patients with a single day of negative FUBCs showed similar diagnostic characteristics (Supplementary Figure 2 and Supplementary Table 3).

**Figure 2.**
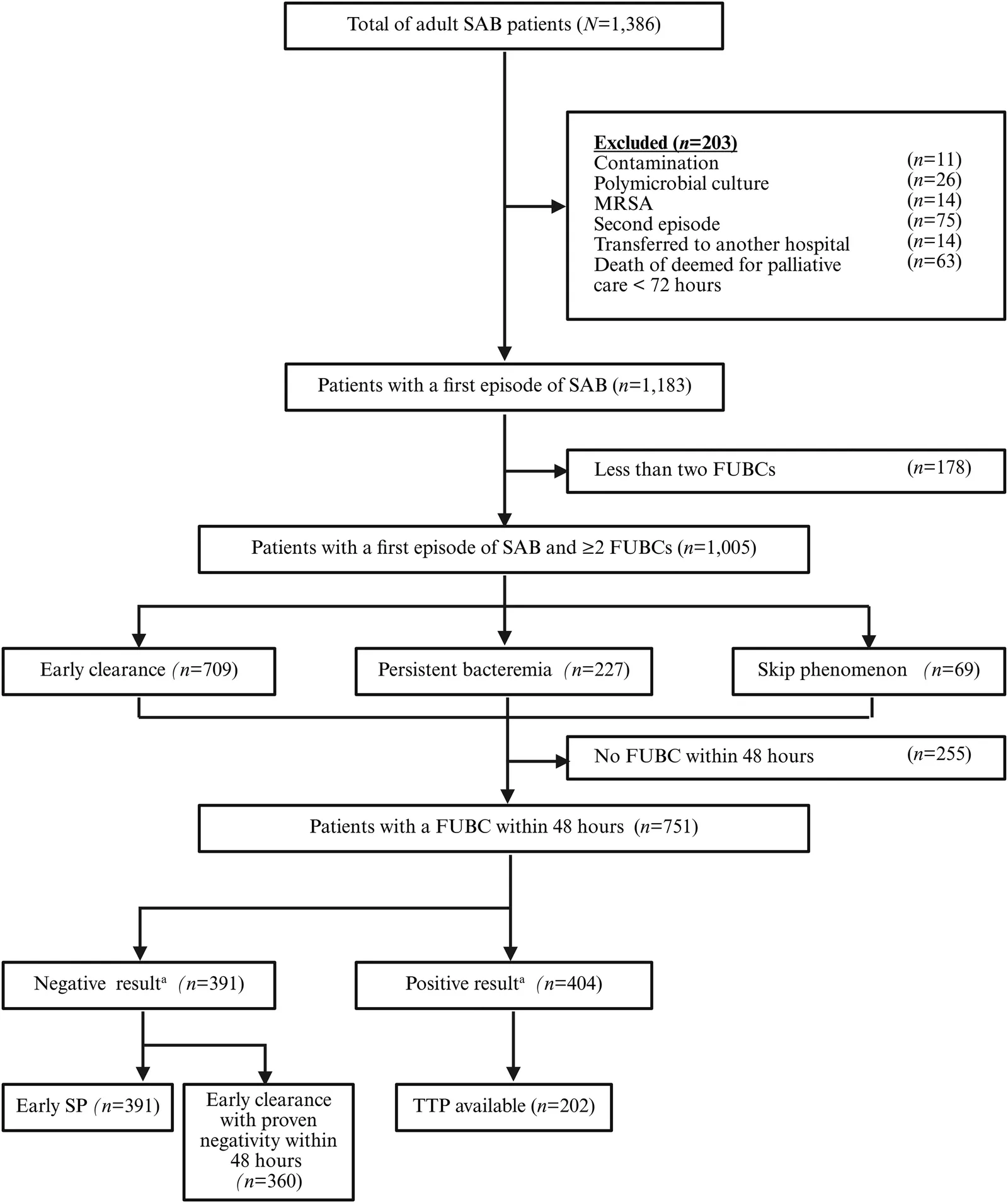
Flowchart of patient inclusion. ^a^Patients are not mutually exclusive. Abbreviations: FUBC, follow-up blood cultures; MRSA, methicillin resistant *Staphylococcus aureus*; SAB, *Staphylococcus aureus* bacteremia.

**Figure 3.**
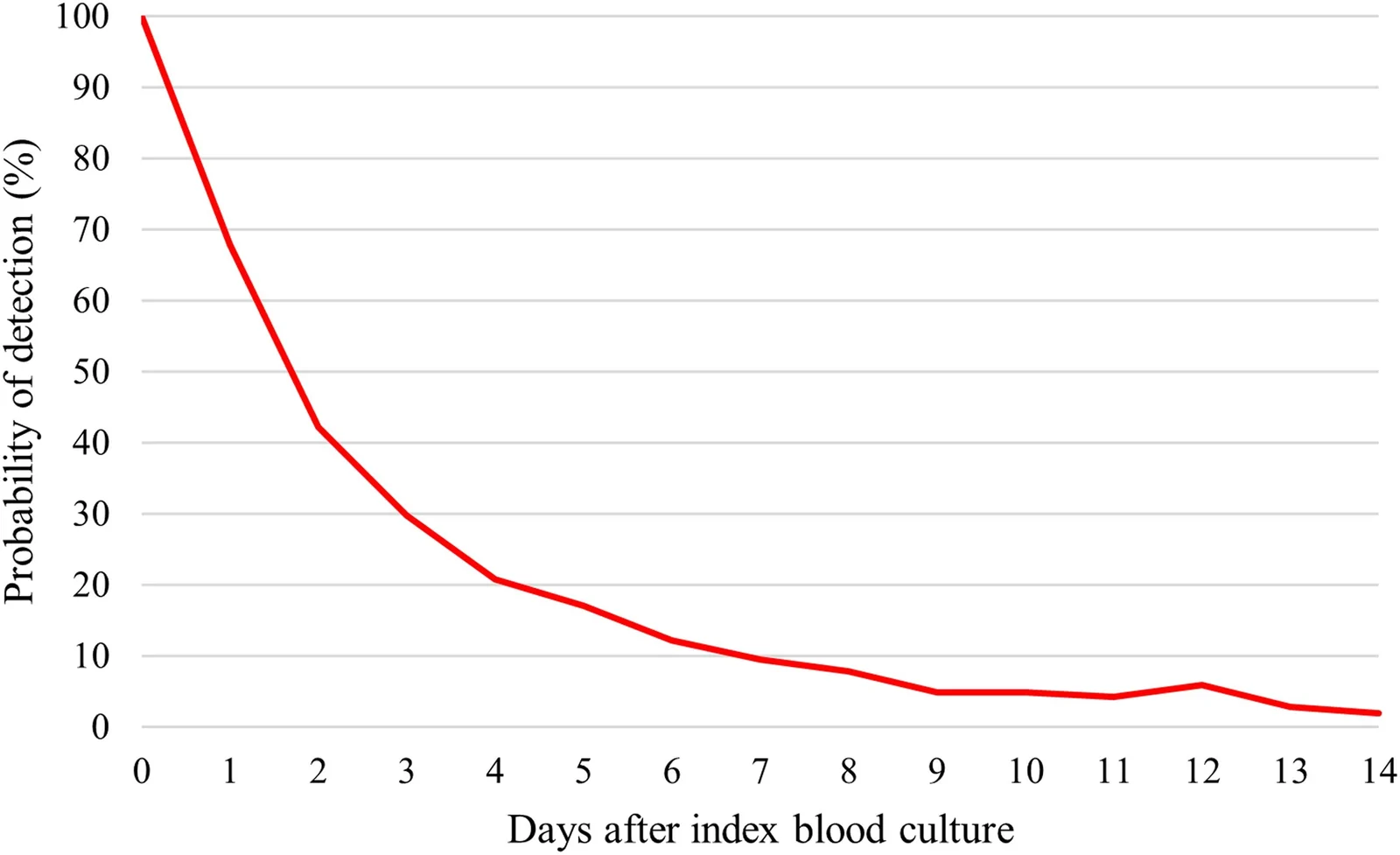
Probability of detecting SAB (y-axis) for each day after the index blood culture (x-axis), based on the number of BC bottles taken and the positivity rate. Abbreviations: SAB, *Staphylococcus aureus* bacteremia; BC, blood culture.

**Table 2.** Follow-up Blood Culture Results in 1005 Patients With SAB During the First 14 Days

Day	Patients With Collected BCs	Accuracy (%)	Sensitivity (%)	Positive BCs (%)	True Negative BCs (%)	False Negative BCs (%)	Predictive Value Of Negative BCs
1	329	97.6	96.5	223 (67.8)	98 (29.8)	8 (2.4)	0.92
2	533	95.7	90.7	225 (42.2)	285 (53.5)	23 (4.3)	0.93
3	458	97.6	92.5	136 (29.7)	311 (67.9)	11 (2.4)	0.97
4	545	97.1	87.6	113 (20.7)	416 (76.3)	16 (2.9)	0.96
5	429	98.4	91.3	73 (17.0)	349 (81.4)	7 (1.6)	0.98
6	427	98.1	86.7	52 (12.2)	367 (85.9)	8 (1.9)	0.98
7	327	98.8	88.6	31 (9.5)	292 (89.3)	4 (1.2)	0.99
8	269	98.9	87.5	21 (7.8)	245 (91.1)	3 (1.1)	0.99
9	229	97.8	68.8	11 (4.8)	213 (93.0)	5 (2.2)	0.98
10	163	99.4	88.9	8 (4.9)	154 (94.5)	1 (0.6)	0.99
11	164	98.2	70.0	7 (4.3)	154 (93.9)	3 (1.8)	0.98
12	120	100.0	100.0	7 (5.8)	113 (94.2)	0 (0)	1.00
13	108	100.0	100.0	3 (2.8)	105 (97.2)	0 (0)	1.00
14	101	100.0	100.0	2 (2.0)	99 (98.0)	0 (0)	1.00

Abbreviations: SAB: *Staphylococcus aureus* bacteremia; BC, blood culture.

Patients with SP and PB showed similar clinical characteristics concerning proportion of community onset, median peak CRP concentrations, metastatic SAB, and all-cause 90-day mortality (Table 3). Positive FUBCs within 48 hours were available in 404 patients. Among these positive FUBCs, 50.0% had a TTP reported with a median TTP of 20 hours (IQR 16–33 hours). In 88.6% of these FUBCs, the result became positive within 48 hours, indicating that the positive result would have been available before collection of the subsequent FUBC in the vast majority of patients. Median TTP of the FUBC within 48 hours did not differ between groups (Table 3, Supplementary Figure 1).

**Table 3.** Patient and Disease Characteristics of Patients Classified as Early Clearance (EC), Persistent Bacteremia (PB), or Skip Phenomenon (SP)

Patient Characteristics	Patients With EC (n = 709)	Patients With PB (n = 227)	Patients With SP (n = 69)	*P* Value
Male	446 (62.9)	145 (63.9)	48 (69.6)	.545
Age (median + IQR), y	68 (54–76)	68 (56–77)	67 (60–74)	.423
Site of bacteremia onset			…	<.001
Community-acquired	237 (33.4)	131 (57.7)	44 (63.8)	<.001
Healthcare-associated	202 (28.5)	46 (20.3)	9 (13.0)	.002
Hospital-acquired	270 (38.1)	50 (22.0)	16 (23.2)	<.001
One positive blood culture bottle at index^a^	128 (18.1)	10 (4.4)	4 (5.8)	<.001
Number of FUBCs (median + IQR)	3 (3–5)	6 (4–7)	7 (5–9)	<.001
Portal of entry			…	<.001
Skin	213 (30.0)	65 (28.6)	23 (33.3)	.753
Peripheral intravenous line related phlebitis	143 (20.2)	26 (11.5)	8 (11.6)	.004
Central venous catheter	115 (16.2)	20 (8.8)	6 (8.7)	.008
Pulmonary	11 (1.6)	2 (0.9)	0 (0)	.455
Other	68 (9.6)	16 (7.0)	6 (8.7)	.504
Unknown entry	159 (22.4)	98 (43.2)	26 (37.7)	<.001
Charlson comorbidity index (median + IQR)	4 (2–6)	4 (2–6)	3 (3–6)	.822
Prosthetic material^b^	308 (43.4)	107 (47.1)	24 (34.8)	.188
Osteoarticular	134 (18.9)	51 (22.5)	13 (18.8)	.492
Intravascular	86 (12.1)	33 (14.5)	5 (7.2)	.260
Cardiac devices	64 (9.0)	26 (11.5)	5 (7.2)	.448
Cardiac valves	52 (7.3)	17 (7.5)	5 (7.2)	.996
Other	42 (5.9)	8 (3.5)	1 (1.4)	.130
Disease characteristics			…	
Bacteremia duration (median + IQR), d	0 (0–1)	4 (3–6)	6 (4–9)	<.001
TTP of FUBCs within 48 h (median + IQR), h^a^	23 (16–37)	19 (16–25)	19 (11–34)	.206
Peak CRP within 48 h (median + IQR), mg/L^a^	153 (82–254)	258 (176–346)	279 (169–332)	<.001
Persistent fever over 72 h^a^	152 (21.9)	82 (36.8)	26 (40.0)	<.001
Metastatic SAB	291 (41.0)	182 (80.2)	51 (73.9)	<.001
Relapse-free-survival 30 d	634 (89.4)	184 (81.1)	55 (79.7)	<.001
30-d mortality	70 (9.9)	41 (18.1)	14 (20.3)	<.001
Reinfection	5 (0.7)	2 (0.9)	0 (0)	.742
Relapse-free-survival 90 d	591 (83.4)	159 (70.0)	44 (63.8)	<.001
90-d mortality	106 (15.0)	64 (28.2)	24 (34.8)	<.001
Reinfection	12 (1.7)	4 (1.8)	1 (1.4)	.985

All data are absolute numbers (%) unless shown otherwise.

Abbreviations: CRP, C-reactive protein; EC, early clearance; FUBC, follow-up blood culture; IQR, interquartile range; PB, persistent bacteremia; SP, skip phenomenon; TTP, time-to-positivity; SAB, *Staphylococcus aureus* bacteremia.

^a^CRP values were available for 665 (93.8%) of EC, 214 (94.3%) of PB, and 66 (95.7%) of SP patients; TTP for 102 (14.3%), 83 (36.6%), and 17 (24.6%); and persistent fever >72 h for 695 (98.4%), 223 (98.2%), and 65 (94.2%), respectively.

^b^Prosthetic material presence is not mutually exclusive.

### Early SP

Thirty-one patients had early SP (3.1%). Compared to EC patients with negative FUBCs within 48 hours, patients with early SP had higher peak CRP values within 48 hours after index blood culture (207 [134–339] mg/L versus 153 [82–254] mg/L, *P* = .005) and higher all-cause 90-day mortality (29.0% vs 15.0%, *P* = .034). No patients with early SP had only 1 single positive BC bottle at index, whereas 18.1% of EC patients did (*P* = .030) (Table 4).

**Table 4.** Patient and Disease Characteristics of Patients Classified as Early Clearance (EC) or Early Skip Phenomenon (SP)

Patient Characteristics	Patients With Early SP (n = 31)	Patients With EC (n = 709)	*P* Value
Male	20 (64.5)	446 (62.9)	.856
Age (median + IQR), y	67 (59–74)	68 (55–76)	.656
Site of bacteremia onset			.049
Community-acquired	17 (54.8)	237 (33.4)	.014
Healthcare-associated	6 (19.4)	202 (28.5)	.268
Hospital-acquired	8 (25.8)	270 (38.1)	.167
One positive blood culture bottle at index^a^	0 (0)	128 (18.1)	.030
Number of FUBCs, median (IQR)	7 (6–10)	3 (3–5)	<.001
Portal of entry			.801
Skin	11 (35.5)	213 (30.0)	.519
Peripheral intravenous line related phlebitis	4 (12.9)	143 (20.2)	.321
Central venous catheter	4 (12.9)	115 (16.2)	.623
Pulmonary	0 (0)	11 (1.6)	.485
Other	3 (9.7)	68 (9.6)	.987
Unknown entry	9 (29.0)	159 (22.4)	.390
Charlson comorbidity index (median + IQR)	4 (3–7)	4 (2–6)	.562
Prosthetic material^b^	12 (38.7)	308 (43.4)	.603
Osteoarticular	8 (25.8)	134 (18.9)	.339
Intravascular	0 (0)	86 (12.1)	.039
Cardiac devices	2 (6.5)	64 (9.0)	.622
Cardiac valves	3 (9.7)	52 (7.3)	.626
Other	1 (3.2)	42 (5.9)	.530
Disease characteristics			
Bacteremia duration (median + IQR), d	4 (4–6)	0 (0–1)	<.001
TTP of FUBCs within 48 h (median + IQR), h^a^	22 (14–59)	23 (16–36)	.940
Peak CRP within 48 h (median + IQR), mg/L^a^	207 (134–339)	153 (82–254)	.005
Persistent fever over 72 h^a^	13 (44.8)	152 (21.9)	.004
Metastatic SAB	18 (58.1)	291 (41.0)	.060
Relapse-free-survival 30 d	26 (83.9)	634 (89.4)	.330
30-d mortality	5 (16.1)	70 (9.9)	.259
Relapse/reinfection	0 (0)	5 (0.7)	.639
Relapse-free-survival 90 d	22 (71.0)	591 (83.4)	.073
90-d mortality	9 (29.0)	106 (15.0)	.034
Relapse/reinfection	0 (0)	12 (1.7)	.465

All data are absolute numbers (%) unless shown otherwise.

Abbreviations: CRP, C-reactive protein; EC, early clearance; FUBC, follow-up blood culture; IQR, interquartile range; SP, skip phenomenon; TTP, time-to-positivity; SAB, *Staphylococcus aureus* bacteremia.

^a^CRP values were available for 31 (100%) of early SP and 665 (93.8%) of EC patients; TTP for 4 (12.9%) and 102 (14.4%); and persistent fever >72 h for 29 (93.5%) and 695 (98.0%), respectively.

^b^Prosthetic material presence is not mutually exclusive.

As shown in Table 5, 87.1% of patients with early SP had a clinical indication for additional FUBCs after their first negative FUBC result, most commonly due to persistent or recurrent fever (61.3%). Among the patients with early SP, FUBCs after the first negative blood culture result were not clinically indicated in only 4 patients. The estimated proportion of potentially missed PB was 1.0% (4/391). A sensitivity analysis by excluding patients with only 1 day of negative FUBCs showed a similar proportion of potentially missed PB (1.0%, 4/385) (Supplementary Figure 2).

**Table 5.** Profile of the Early Skip Phenomenon Patients

Number of patients	31 (3.1)
Number of blood culture sets performed on the day with SP, median (IQR)	2 (1–2)
Reasons for performing additional follow-up blood cultures after SP	…
Persistent fever	14 (45.2)
Recurrent fever	5 (16.1)
ICU admission	1 (3.2)
Late source control	3 (9.7)
Confirmed metastatic infection	4 (12.9)
Routine follow-up	4 (12.9)
Number of positive blood culture bottles on the day after SP, median (IQR)	2 (2–4)
1 out of 2 bottles	5 (16.1)
1 out of 4 bottles	13 (41.9)
2 out of 4 bottles	4 (12.9)
2 out of 2 bottles	4 (12.9)
4 out of 4 bottles	5 (16.1)
Interval to subsequent positive blood cultures, days, median (IQR)	2 (1–3)
Interval to the last positive blood cultures, days, median (IQR)	2 (2–4)

Data are presented as n (%) or median (interquartile range, IQR).

Abbreviations: ICU, intensive care unit; IQR, interquartile range; SP, skip phenomenon.

### Costs

In this cohort, a total of 10 510 blood cultures were obtained from 1005 patients in the first 14 days following SAB during hospitalization. This corresponded to a total cost of 389 009 USD. Discontinuing routine FUBC without a clinical indication after a day of negative FUBCs within 48 hours of SAB onset would have prevented 577 additional blood culture sets across 166 patients. The estimated costs of unnecessary FUBCs during the study period was 22 431 USD, corresponding with a potential cost saving of 13 513 USD per 100 patients with a day of negative FUBCs within 48 hours after SAB onset.

## DISCUSSION

In this multicenter observational study, early SP in SAB was uncommon, with an overall prevalence of 3.1%. An early intermittent negative blood culture may falsely suggest low risk SAB, potentially leading to inadequate source evaluation, or premature de-escalation or shortening of therapy. However, nearly all early SP cases (27 of 31) were detected because patient's clinical condition prompted further FUBCs. If a single day of negative FUBCs within 48 hours after SAB onset had been considered sufficient, 4 patients without evident clinical triggers would have been misclassified as having EC. Therefore, clinically stable, afebrile patients without signs of metastatic infection who have a single day of negative FUBCs within 48 hours have a very low risk of PB being missed (1.0%).

The proportion of patients in our study with SP (6.9%) is in line with previous studies, ranging from 4% to 13% [7–12]. None of these studies distinguished early SP from late SP, which is a significant limitation. Only Jang et al (2025) described SP in PB, which can be classified as late SP [8]. However, the only clinical implication of late SP is its potential influence on treatment duration, as antibiotic therapy length is determined by the timing of the first negative FUBC [8]. Given that treatment for complicated SAB typically extends for generally 6 weeks, the clinical relevance of a delay of only a few days is debatable.

Due to the risk of SP, previous studies have recommended taking 2 FUBC sets on day 2 and 4 after the start of therapy to detect ≥90% of persisting SAB, irrespective of clinical indications [7, 11]. Based on our findings, we propose performing a FUBC set at day 2 in all patients and tailoring subsequent cultures according to this result and clinical indications. If the day-2 FUBCs are negative and after another 2 days there are no clinical triggers, such as persistent or recurrent fever, clinical deterioration, suspicion of metastatic infection, or intravascular- or intracardiac-prosthetic material in situ, we strongly recommend discontinuing routine FUBCs. This recommendation is supported by the TTP of the first FUBC, which was <48 hours in almost all patients, indicating that it is safe to adopt a “wait-and-see” approach and base further decisions on clinical and microbiological factors. This approach also generates substantial cost savings, as unnecessary FUBCs accounted for an estimated cost of 22 431 USD in this subgroup. Although current studies do not recommend obtaining more than 2 days of negative FUBCs, this practice was common in our cohort and likely reflects real-world practice in other high-income healthcare settings [2, 10]. Nevertheless, in an era of escalating healthcare expenditures driven by population aging, multimorbidity, and rising costs, strategies that safely reduce unnecessary interventions are essential. Streamlining FUBC practices not only minimizes waste but also optimizes resource allocation without significantly compromising patient safety.

Strengths of this study include its multicenter design, the specific focus on early SP, the identification of clinical triggers for FUBCs after early SP or EC, and the evaluation of potential cost savings associated with streamlining FUBC practices. This study has also limitations, primarily related to its retrospective design. First, reasons for SP could not be assessed. Blood culture sensitivity is strongly influenced by collection volume [20, 21], which was not available in our study. Second, timing of FUBC relative to antimicrobial therapy was not recorded; cultures drawn immediately after antimicrobial administration may have reduced sensitivity. However, this still represents clinical practice in real life. Third, our study was underpowered to identify predictors of early SP. Fourth, exclusion of MRSA may limit generalizability to patients with MRSA bacteremia. Fifth, exclusion of patients who did not have at least 2 days of FUBCs (15%) may have introduced bias. However, this was necessary to reliably diagnose SP. Sixth, our cost analysis was restricted to patients with documented negative FUBCs within 48 hours, thereby excluding those whose first negative FUBC was performed later. Only 55% of EC patients met this criterion. Because many of the remaining EC patients would likely have had negative FUBCs within 48 hours if cultures had been obtained, this restriction may have substantially underestimated the potential cost savings. Seventh, although our practical approach resulted in substantial cost savings, the clinical and economic consequences of missing PB could outweigh these savings. While our data indicate that the risk of missing PB in this subgroup is very low, the potential impact of such missed cases should be considered when interpreting the implications of our proposed strategy.

In conclusion, our findings support an efficient and practical approach: obtain a FUBC set at day 2, and base further cultures on clinical and microbiological factors rather than perform FUBCs as a routine practice. This strategy reduces unnecessary interventions, minimizes costs, and optimizes resource utilization without significantly compromising patient safety.

## Supplementary Material

ciag418_Supplementary_Data

## Supplementary Data

Supplementary materials are available at *Clinical Infectious Diseases* online. Consisting of data provided by the authors to benefit the reader, the posted materials are not copyedited and are the sole responsibility of the authors, so questions or comments should be addressed to the corresponding author.

## References

[1] Fowler VG, Jr., Olsen MK, Corey GR, et al Clinical identifiers of complicated Staphylococcus aureus bacteremia. Arch Intern Med 2003; 163:2066–72. 10.1001/archinte.163.17.2066 14504120

[2] Kuehl R, Morata L, Boeing C, et al Defining persistent Staphylococcus aureus bacteraemia: secondary analysis of a prospective cohort study. Lancet Infect Dis 2020; 20:1409–17. 10.1016/S1473-3099(20)30447-3 32763194

[3] Liu C, Bayer A, Cosgrove SE, et al Clinical practice guidelines by the Infectious Diseases Society of America for the treatment of methicillin-resistant Staphylococcus aureus infections in adults and children. Clinical Infectious Diseases 2011; 52:e18–55. 10.1093/cid/ciq146 21208910

[4] van der Vaart TW, Prins JM, Soetekouw R, et al All-cause and infection-related mortality in Staphylococcus aureus bacteremia, a multicenter prospective cohort study. Open Forum Infect Dis 2022; 9:ofac653. 10.1093/ofid/ofac653 PMC9792080 36589483

[5] Kouijzer IJE, Fowler VG, Jr., Ten Oever J. Redefining Staphylococcus aureus bacteremia: a structured approach guiding diagnostic and therapeutic management. J Infect 2023; 86:9–13. 10.1016/j.jinf.2022.10.042 PMC11105116 36370898

[6] Verduin K, Ammerlaan H, Blaauw G, et al Dutch guidelines on the treatment of *Staphylococcus aureus* bacteremia. 2019. Available at: https://richtlijnendatabase.nl/richtlijn/staphylococcus_aureus_bacteriemie/startpagina.html. Accessed 10 November 2025.

[7] Fiala J, Palraj BR, Sohail MR, Lahr B, Baddour LM. Is a single set of negative blood cultures sufficient to ensure clearance of bloodstream infection in patients with Staphylococcus aureus bacteremia? The skip phenomenon. Infection 2019; 47:1047–53. 10.1007/s15010-019-01339-w PMC7102132 31297719

[8] Jang S, Jeon M, Kim S-H, Mun SJ. Clinical implications of the skip phenomenon in patients with persistent *Staphylococcus aureus* bacteremia. Microb Drug Resist 2025; 31:21–5. 10.1089/mdr.2024.0107 39632763

[9] Go JR, Baddour LM, Lahr BD, Sohail MR, Palraj R. The skip phenomenon in Staphylococcus aureus bacteremia: clinical implications. Diagn Microbiol Infect Dis 2022; 104:115802. 10.1016/j.diagmicrobio.2022.115802 36137340

[10] Stewart JD, Graham M, Kotsanas D, Woolley I, Korman TM. Intermittent negative blood cultures in *Staphylococcus aureus* bacteremia; a retrospective study of 1071 episodes. Open Forum Infect Dis 2019; 6:ofz494. 10.1093/ofid/ofz494 PMC7047948 32128336

[11] Van Goethem S, Boogaerts H, Cuykx M, et al Follow-up blood cultures in Staphylococcus aureus bacteremia: a probability-based optimization. Eur J Clin Microbiol Infect Dis 2022; 41:1263–8. 10.1007/s10096-022-04487-4 36066759

[12] Cardenas-Comfort C, Kaplan SL, Vallejo JG, McNeil JC. Follow-up blood cultures in children with *Staphylococcus aureus* bacteremia. Pediatrics 2020; 146:e20201821. 10.1542/peds.2020-1821 33239473

[13] Go JR, Challener D, Campioli C, et al Clinical significance of Staphylococcus aureus in a single positive blood culture bottle. Open Forum Infect Dis 2021; 9:ofab642. 10.1093/ofid/ofab642 PMC8774077 35071685

[14] Hendriks MMC, Schweren KSA, Kleij A, et al Low-risk Staphylococcus aureus bacteremia patients do not require routine diagnostic imaging: a multicenter retrospective cohort study. Clin Infect Dis 2024; 79:43–51. 10.1093/cid/ciae187 PMC11259217 38576380

[15] van Cleef BAGL, Kluytmans JAJW, van Benthem BHB, et al Cross border comparison of MRSA bacteraemia between The Netherlands and North Rhine-Westphalia (Germany): a cross-sectional study. PLoS One 2012; 7:e42787. 10.1371/journal.pone.0042787 PMC3411841 22880109

[16] Charlson ME, Pompei P, Ales KL, MacKenzie CR. A new method of classifying prognostic comorbidity in longitudinal studies: development and validation. J Chronic Dis 1987; 40:373–83. 10.1016/0021-9681(87)90171-8 3558716

[17] Kim S-H, Jeon M, Jang S, Mun SJ. Factors for mortality in patients with persistent Staphylococcus aureus bacteremia: the importance of treatment response rather than bacteremia duration. J Microbiol Immunol Infect 2023; 56:1007–15. 10.1016/j.jmii.2023.07.010 37580183

[18] Minejima E, Mai N, Bui N, et al Defining the breakpoint duration of Staphylococcus aureus bacteremia predictive of poor outcomes. Clin Infect Dis 2020; 70:566–73. 10.1093/cid/ciz257 PMC7768749 30949675

[19] Canzoneri CN, Akhavan BJ, Tosur Z, Andrade PEA, Aisenberg GM. Follow-up blood cultures in gram-negative bacteremia: are they needed? Clin Infect Dis 2017; 65:1776–9. 10.1093/cid/cix648 29020307

[20] Miller JM, Binnicker MJ, Campbell S, et al A guide to utilization of the microbiology laboratory for diagnosis of infectious diseases: 2018 update by the Infectious Diseases Society of America and the American Society for Microbiology. Clin Infect Dis 2018; 67:e1–94. 10.1093/cid/ciy381 PMC7108105 29955859

[21] Khare R, Kothari T, Castagnaro J, Hemmings B, Tso M, Juretschko S. Active monitoring and feedback to improve blood culture fill volumes and positivity across a large integrated health system. Clin Infect Dis 2020; 70:262–8. 10.1093/cid/ciz198 30873522

