# Supplementary material for: Routine Serial Follow-up Blood Cultures After a Negative Result Within 48 Hours of Methicillin-susceptible Staphylococcus aureus Bacteremia: Low Yield, High Cost
Source: Clin Infect Dis. 2026 Jul 15;83(4):e745–53. doi: 10.1093/cid/ciag418 (PMC13715978; doi:10.1093/cid/ciag418)
Supplement: Supplementary file 1 [file ciag418_supplementary_data.docx]

**Routine serial follow-up blood cultures after a negative result within 48 hours of methicillin-susceptible *Staphylococcus aureus* bacteremia: low yield, high cost**

**Marianne M.C. Hendriks, MD^1,a,#^, Eline H.H.M. Kamps, BSc^1,a^, Cairo Norberhuis, MD^2^, Mihai G. Netea, MD, PhD^1,3^, Matthijs S. Berends, PhD^4,5^, Ianthe Maat, MD^6^, Sander van Assen, MD, PhD^2^, Jaap ten Oever, MD, PhD^1b^, Ilse J.E. Kouijzer, MD, PhD^1b^**

**Supplementary data**

**Table of contents**

| Supplementary Table S1 | Inclusion period, microbiology systems and TTP availability of participating hospitals | 3 |
| --- | --- | --- |
|  |  |  |
| Supplementary Figure S1 | Time-to-positivity distribution of FUBCs within 2 days after index blood culture | 4 |
|  |  |  |
| Supplementary Table S2 | Prevalence of early clearance (EC), persistent bacteremia (PB) and skip phenomenon (SP) in patients with or without intravascular- and intracardial prosthetic material | 5 |
|  |  |  |
| Supplementary Figure S2 | Flowchart of patient inclusion. Sensitivity analysis including only patients with ≥2 days of negative FUBCs if negativity was reached | 6 |
|  |  |  |
| Supplementary Table S3 | Sensitivity analysis including only FUBC results in 936 patients with SAB with sustained negativity during the first 14 days | 7 |
|  |  |  |
|  |  |  |

**Supplementary Table S1.** Inclusion period, microbiology systems and TTP availability of participating hospitals

| Hospital | Hospital characteristics | Inclusion period | Microbiology system | TTP available from |
| --- | --- | --- | --- | --- |
| Hospital 1 | University hospital | 01-2013 until 03-2025 | BD BACTEC^TM^ | 04-2017 |
| Hospital 2 | Non-university teaching hospital | 01-2019 until 05-2025 | BacT/ALERT | 10-2023 |

Abbreviations: BD BACTEC: Becton, Dickinson and Company Bacteria Environmental Culture system; BacT/ALERT: Bacteria alert; TTP: time-to-positivity

**Supplementary Figure S1.** Time-to-positivity distribution of FUBCs within 48 hours after index blood culture

Time-to-positivity (TTP) data of positive follow-up blood cultures (FUBCs) obtained within 48 hours after the index culture were available for 404 patients. The median TTP was 20 hours (IQR: 16-33) and 179 FUBCs (88.6%) had a TTP of less than 48 hours, indicating that the positive result would have been known before the subsequent FUBC was collected in the majority of patients.

**Supplementary Table S2**. Prevalence of early clearance (EC), persistent bacteremia (PB) and skip phenomenon (SP) in patients with or without intravascular- and intracardial prosthetic material

| **Classification** | **Patients with intravascular- or intracardiac prosthetic material (n= 243)** | **Patients without intravascular- or intracardiac prosthetic material (n=762)** | ***p*-value** |
| --- | --- | --- | --- |
| Early clearance | 168 (69.1) | 540 (70.9) | .607 |
| Persistent bacteremia | 62 (25.5) | 166 (21.8) | .227 |
| Skip phenomenon | 13 (5.3) | 56 (7.3) | .283 |

Total of adult SAB patients (*N*=1386)

**Excluded (*n*=203)**

Contamination	(*n*= 11)

Polymicrobial culture	(*n*= 26)

MRSA	(*n*= 14)

Second episode	(*n*= 75)

Transferred to another hospital	(*n*= 14)

Death or deemed for palliative	(*n*= 63)

care <72 hours

Patients with a first episode of SAB (*n*=1183)

Less than two days of FUBCs	(*n*= 178)

Patients with a first episode of SAB and ≥2 FUBCs (*n*=1005)

Only one day of negative FUBCs	(*n*= 69 )

Patients with a first episode of SAB and ≥2 FUBCs and -if negativity was reached- ≥2 negative FUBC (*n*=936)

Skip phenomenon (*n*=55)

Persistent bacteremia (*n*=204)

Early clearance (*n*=677 )

No FUBC within 48 hours	(*n*= 238)

Patients with a FUBC result within 48 hour (*n*=698)

Positive result^a^ (*n*=356)

Negative result^a^ (*n*= 385)

Sustained negativity (*n*=358) (n=181)

Early SP (*n*=27)

Clinical indication for FUBC (*n*=23)

Routine FUBC (*n*=4)

**Supplementary Figure S2.** Flowchart of patient inclusion. Sensitivity analysis including only patients with ≥2 negative FUBCs if negativity was reached

*^a^ Patients are not mutually exclusive*

Abbreviations: SAB: *Staphylococcus aureus* bacteremia, MRSA: methicillin resistant *Staphylococcus aureus*, FUBC: follow-up blood cultures; SP: Skip phenomenon

**Supplementary Table S3.** Sensitivity analysis including only follow-up blood culture results in 936 patients with SAB with sustained negativity during the first 14 days

| Day | Patients with Collected BCs | Accuracy  (%) | Sensitivity  (%) | True Positive BCs (%) | True Negative BCs (%) | False Negative BCs (%) | Predictive Value of Negative BCs |
| --- | --- | --- | --- | --- | --- | --- | --- |
| 1 | 304 | 97.7 | 96.6 | 200 (65.8) | 97 (31.9) | 7 (2.3) | 0.93 |
| 2 | 499 | 96.0 | 90.8 | 197 (39.5) | 282 (56.5) | 20 (4.0) | 0.93 |
| 3 | 435 | 97.9 | 93.2 | 123 (28.3) | 303 (69.7) | 9 (2.1) | 0.97 |
| 4 | 516 | 97.7 | 89.0 | 97 (18.8) | 407 (78.9) | 12 (2.3) | 0.97 |
| 5 | 410 | 99.0 | 94.2 | 65(15.9) | 341 (83.2) | 4 (1.0) | 0.99 |
| 6 | 403 | 98.8 | 90.0 | 45 (11.2) | 353 (87.6) | 5 (1.2) | 0.99 |
| 7 | 313 | 98.7 | 86.2 | 25 (8.0) | 284 (90.7) | 4 (1.3) | 0.99 |
| 8 | 265 | 99.6 | 95.5 | 21 (7.9) | 243 (91.7) | 1 (0.4) | 1.00 |
| 9 | 214 | 99.1 | 77.8 | 7 (3.3) | 205 (95.8) | 2 (0.9) | 0.99 |
| 10 | 162 | 99.4 | 87.5 | 7 (4.3) | 154 (95.1) | 1 (0.6) | 0.99 |
| 11 | 162 | 98.1 | 62.5 | 5 (3.1) | 154 (95.1) | 3 (1.9) | 0.98 |
| 12 | 119 | 100.0 | 100.0 | 5 (4.2) | 114 (95.8) | 0 (0) | 1.00 |
| 13 | 106 | 100.0 | 100.0 | 3 (2.8) | 103 (97.2) | 0 (0) | 1.00 |
| 14 | 97 | 100.0 | 100.0 | 2 (2.1) | 95 (97.9) | 0 (0) | 1.00 |

Abbreviations: SAB, *Staphylococcus aureus* bacteremia; BC, blood culture.
